# Predicting the Severity of Neurological Impairment Caused by Ischemic Stroke Using Deep Learning Based on Diffusion-Weighted Images

**DOI:** 10.3390/jcm11144008

**Published:** 2022-07-11

**Authors:** Ying Zeng, Chen Long, Wei Zhao, Jun Liu

**Affiliations:** 1 Department of Radiology, The Second Xiangya Hospital, Central South University, Changsha 410011, China; zy6645295@163.com; 2 Department of Radiology, Xiangtan Central Hospital, Xiangtan 411199, China; 3 Department of Stroke Unit, Xiangtan Central Hospital, Xiangtan 411199, China; long_ali@tom.com; 4 Clinical Research Center for Medical Imaging, Changsha 410011, China; 5 Department of Radiology Quality Control Center, Changsha 410011, China

**Keywords:** ischemic stroke, convolutional neural networks, NIHSS

## Abstract

**Purpose:** To develop a preliminary deep learning model that uses diffusion-weighted imaging (DWI) images to classify the severity of neurological impairment caused by ischemic stroke. **Materials and Methods:** This retrospective study included 851 ischemic stroke patients (711 patients in the training set and 140 patients in the test set). The patients’ NIHSS scores, which reflect the severity of neurological impairment, were reviewed upon admission and on Day 7 of hospitalization and were classified into two stages (stage 1 for NIHSS < 5 and stage 2 for NIHSS ≥ 5). A 3D-CNN was trained to predict the stage of NIHSS based on different preprocessed DWI images. The performance in predicting the severity of anterior and posterior circulation stroke was also investigated. The AUC, specificity, and sensitivity were calculated to evaluate the performance of the model. **Results:** Our proposed model obtained better performance in predicting the NIHSS stage on Day 7 of hospitalization than that at admission (best AUC 0.895 vs. 0.846). Model D trained with DWI images (normalized with z-score and resized to 256 × 256 × 64 voxels) achieved the best AUC of 0.846 in predicting the NIHSS stage at admission. Model E rained with DWI images (normalized with maximum–minimum and resized to 128 × 128 × 32 voxels) achieved the best AUC of 0.895 in predicting the NIHSS stage on Day 7 of hospitalization. Our model also showed promising performance in predicting the NIHSS stage on Day 7 of hospitalization for anterior and posterior circulation stroke, with the best AUCs of 0.905 and 0.903, respectively. **Conclusions:** Our proposed 3D-CNN model can effectively predict the neurological severity of IS using DWI images and performs better in predicting the NIHSS stage on Day 7 of hospitalization. The model also obtained promising performance in subgroup analysis, which can potentially help clinical decision making.

## 1. Introduction

Ischemic stroke (IS), which has high mortality and disability rates [1], is the most common type of stroke and places a heavy burden on patients’ families, as well as society in general [2]. According to the guidelines for the management of patients with IS, timely medical intervention is the key treatment to reduce brain tissue injury as much as possible. Accurately and timely evaluating the severity of IS is of great significance in guiding the treatment scenario and facilitating early medical intervention [3].

The National Institutes of Health Stroke Scale (NIHSS), the most common neurological scale used worldwide to assess the severity of IS, is generally used to help inform clinical treatment decisions and predict the outcome with high validity and reliability [4,5]. The NIHSS includes 11 items (consciousness, gaze, visual fields, sensory, language, etc.), and each item is assigned a score of 0–2, 0–3, or 0–4 points. The maximum score is 42. Previous studies have verified that patients with an NIHSS score of 5 have poorer short-term outcomes than patients with an NIHSS score of 4 or less [6]. Therefore, the correct classification of the NIHSS stage can guide clinical treatment decisions, especially for reperfusion treatment. However, the assessment of NIHSS is time-consuming and relatively subjective. A more accurate, efficient, and objective way to evaluate the severity of IS is urgently needed in clinical practice.

Medical imaging plays a vital role in the management of IS patients, and the preferred modality is non-contrast CT, which is mainly applied to exclude hemorrhage and identify conditions other than angiopathy, such as brain tumors and intracranial infections. The Alberta Stroke Program Early CT Score (ASPECTS) is usually evaluated during the hyperacute stage before thrombolytic therapy in order to assess the risk of symptomatic intracranial hemorrhage and the functional prognosis of IS [7]. This scale has a sensitivity of 78% and a specificity of 96% in predicting functional outcomes. This means that the ASPECTS has a relatively low sensitivity. Diffusion-weighted imaging (DWI) sequencing is a first-line diagnostic tool for acute ischemic stroke [8] and has a high specificity and sensitivity in the detection of ischemic lesions [8]. Previously published studies have reported that the DWI infarct volume can be used to predict neurological severity and is correlated with NIHSS score [9,10]. However, the accurate segmentation of the DWI infarct volume is relatively time-consuming and subjective. Sometimes the DWI infarct volume may face the disadvantage of clinical–diffusion mismatch [11]. Therefore, a new potential solution is needed to evaluate the most valuable part of DWI images that can reflect the severity of IS.

At present, the information from radiological images mainly relies on visual scores, such as ASPECTS and the collateral score. However, visual scoring may ignore information in images with millions of pixels. Meanwhile, the application of the scales results in differences in observation among different observers, which affects the reliability of the scales. In recent years, convolutional neural networks (CNNs) have emerged as one of the most prominent methods for medical image analysis, such as classification [12], segmentation [13], and prediction [14]. CNNs can potentially evaluate invisible features and learn internal biological information, which could help in clinical decision making. In some specific medical tasks, deep learning models have outperformed clinicians [15]. With the development of deep learning, CNNs are also being used to analyze DWI images of ischemic stroke patients for tasks such as detection and vascular territorial classification [16], segmentation of ischemic lesions [17,18], and lesion volume measurement [19]. Recently, several studies have focused on functional outcome prediction using deep learning via DWI images [20]. However, no previous studies have investigated the potential of CNN in predicting the NIHSS score using DWI images.

In this study, a deep learning system was designed to address the issue of automatically classifying the stage of NIHSS (stage 1 for NIHSS < 5 and stage 2 for NIHSS ≥ 5) based on DWI images. The main contributions are as threefold: First, we proposed an automatic 3D-CNN to predict the NIHSS stage at different time points, trained by DWI images and the corresponding NIHSS stage labels. The input DWI images were preprocessed by different voxel sizes and normalization strategies. To the best of our knowledge, this is the first automatic learning system for this problem. Second, we further investigated the performance of our model in predicting the NIHHS stage in different circulation areas to evaluate the efficiency of our model. Third, we collected DWI images from 851 patients, and the experimental results showed that our proposed model can effectively predict the neurological severity of IS using DWI images and performs better in predicting the NIHSS stage on Day 7 of hospitalization. The model also obtained promising performance in subgroup analysis, which can potentially help timely clinical decision making.

In the following four sections, we first introduce the materials and methods, including patient selection and the technical details of the proposed method (Section 2). Then, we report the experimental results (Section 3) and finally discuss the results presented in the current study (Section 4) before drawing a conclusion (Section 5).

## 2. Materials and Methods

**Figure 1:**
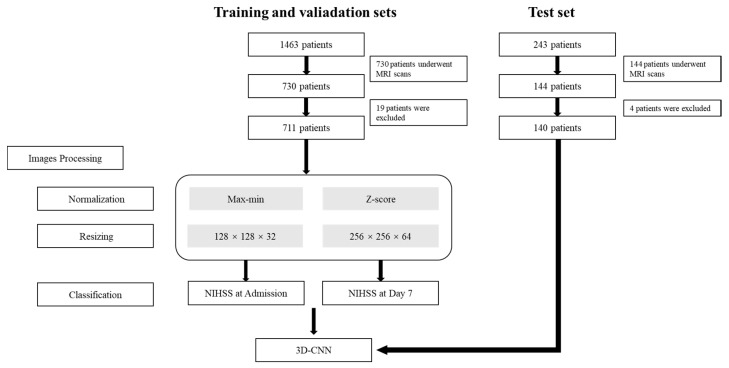
Flow chart of this study. CNN, convolutional neural network, NIHSS, the national institutes of health stroke scale.

This study was approved by the institutional review board of our institution (Approved Number 2021-03-002), which waived the requirement for patients’ informed consent, as this is a retrospective study. The workflow is presented in Figure 1.

### 2.1. Patients

Patients with IS who were admitted to Xiangtan Central Hospital, Hunan Province, from 1 March 2017 to 31 December 2020 were enrolled retrospectively for the training and validation sets. Moreover, patients who were admitted to the same hospital from 1 January 2021 to 1 May 2021 were enrolled for the test set. Patients who underwent MRI scans from 24 h to 7 days after stroke onset in the subacute period were enrolled in order to avoid the influence of ischemic penumbra, which could lead to mismatching between DWI images and NIHSS scores. These patients received intravenous thrombolysis by rt-PA (recombinant tissue plasminogen activator) or urokinase. The following exclusion criteria were applied: (1) an MRI scan was performed within 24 h of ischemic stroke onset to determine if ischemic penumbra was present; (2) the patient had a history of neurological impairment; (3) the image quality of the MRI scans was poor; (4) the patient suffered a recurrent stroke; (5) symptomatic intracranial hemorrhage occurred. 

### 2.2. MRI Scanning Protocols

The included patients underwent MRI scans with the following four scanners: two Siemens Magnetom Aera devices, a Philips Achieva 3.0T X-series, and a Siemens Symphony. Brain MRI sequences, including T1-weighted imaging (T1WI), T2-weighted imaging (T2WI), fluid-attenuated inversion recovery (FLAIR), apparent diffusion coefficient (ADC), and DWI, were performed after the hyperacute period.

### 2.3. Classification

The neurological impairment of patients with ischemic stroke is closely related to the location and scope of ischemic lesions. The NIHSS is the most commonly used scale to assess neurological impairment, and the NIHSS score at admission forms the basis of the treatment strategy. Moreover, the NIHSS score on Day 7 of hospitalization has higher validity for long-term neurological impairment assessment. Therefore, we employed 3D-CNN models based on DWI images to evaluate the neurological impairment using NIHSS scores at admission and on Day 7 of hospitalization. We scored the patients’ neurological function with NIHSS scores at the time of hospital admission and repeated the assessment seven days after stroke onset. Patients who scored below 5 on the NIHSS were classified as stage 1, representing minor stroke, and those who scored at least 5 were classified as stage 2 for severe stroke. All NIHSS evaluations were completed by experienced, professionally trained neurologists, each of whom had at least five years of clinical experience in neurology.

### 2.4. Image Preprocessing

In this study, image preprocessing included the following steps: removing the artifacts, position correction (registration), normalization, and resampling.

Digital Imaging and Communications in Medicine (DICOM) DWI data were collected and transformed into the Neuroimage Informatics Technology Initiative (NIFTI) format, and the high intensity of the ischemic lesions on the DWI images ensured that ADC (apparent diffusion coefficient) values were reduced to avoid T2 shine-through effects.

We removed artifacts in the DWI images, especially the high signal caused by an uneven magnetic field, and replaced the value with zero. Then, we corrected the image position to a standard position by 3D-slicer (https://www.slicer.org, accessed on 1 July 2021).

**Figure 2:**
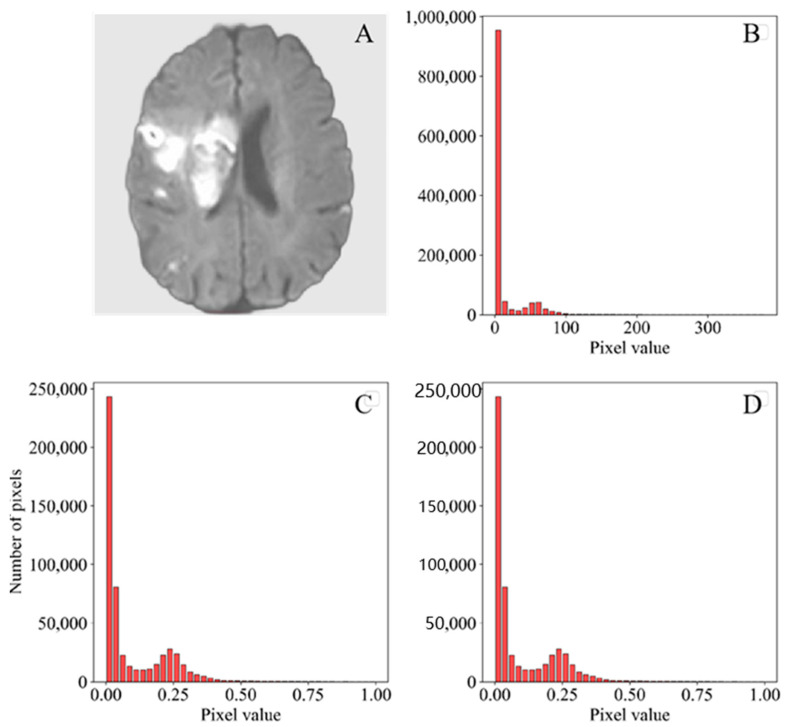
Pixel distribution before and after preprocessing. (**A**) DWI image of ischemic stroke. (**B**) Pixel distribution before preprocessing by the maximum–minimum. (**C**) Pixel distribution after preprocessing by the maximum–minimum. (**D**) Pixel distribution after preprocessing by the z-score.

Since the MRI scans were collected from different MRI scanners, the global signal intensity might be significantly different across machines. To best avoid the effect of different global signal intensities on the models, we performed normalization before model training. The images were normalized using two methods: maximum–minimum and z-score. For the maximum–minimum, the intensity of each voxel was transformed to the range of 0–1 using Equation (1), where *x* is the intensity of each voxel before transition and *x_nor_* is the intensity after the transition. For the z-score, the images were normalized by Equation. (2), where *x_nor_* is the intensity after transition, *x* is the intensity of each pixel, *u* is the mean of the voxel values of all images, and *σ* is the standard deviation of intensity of each voxel (Figure 2).

(1) $$x_{scale}=\left(x-x_{min}\right)/\left(x_{max}-x_{min}\right)$$

(2) $$\;\;\;\;\;\;x_{nor}=\left(x-u\right)\;/\sigma$$

Considering that the original DWI images had different spatial resolutions due to the different scanning parameters between scanners, we resized the images to 128 × 128 × 32 voxels and 256 × 256 × 64 voxels by SimpleITK (https://itk.org, accessed on 15 July 2021) in order to investigate the best voxel size for the current task. The reconstructed voxel values were generated by the linear interpolation algorithm.

### 2.5. Convolutional Neural Network Construction

**Figure 3:**
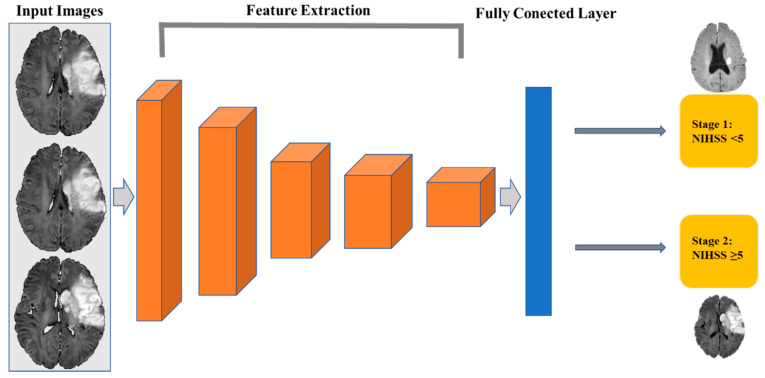
The architecture of the 3D-CNN.

In this section, we used a 3D-CNN based on a VGG net to solve the problem of NIHSS stage classification and described our proposed approach. The framework of our proposed model is presented in Figure 3. DWI images labeled by different NIHSS stages were set as the input. Different voxel sizes and preprocessing strategies were performed to evaluate the influence of data variation. Meanwhile, the NIHSS scores at different time points were tested to avoid overfitting. This is the first study to address this problem.

A 3D-CNN can examine all volumetric information, which is different from the 2D-CNN architecture. We employed a 3D convolutional layer that can extract a feature map exploiting the entire volumetric spatial information, including both local and global contextual information, and can produce a better quality of the local optima [21]. The 3D-CNN was developed in *PyTorch* (version 1.0; Facebook, Menlo Park, CA, USA). The network was built with nine convolutional layers, five maximum pool layers, and three fully connected layers (Figure 3 and Table 1). Data were randomly divided into a training set (569 patients) and a validation set (142 patients) at a ratio of 8:2. Training was performed for 100 epochs with the following parameters: a learning rate of 10^–4^, an adaptive moment estimation (Adam) optimizer, a batch size of eight DWI scans, and a cross-entropy loss function.

The nonlinearity that transforms the data is caused by the activation function. Instead of traditional “hyperbolic tangent” or “sigmoid” functions, we used a rectifier linear unit (ReLU) in this study. On CNN, there were two traditional forms of pooling: maximum pooling [22] and average pooling [23], both of which adopt down-sampling techniques. Due to the significant signal of ischemic lesions on DWI images, maximum pooling was adopted in this investigation, which could result in more substantial responses. To refresh network weights, we used Adam as an optimization tool for the classical stochastic gradient descent (SGD). Adam is a hybrid algorithm that combines the adaptive gradient technique and the root mean square error. We also tested our dataset with different networks, such as Desnet121, Resnet 18, and ResNeXt. The performance of our proposed models outperformed others (Supplemental File).

All computations were performed on NVIDIA GeForce 1070 graphics processing units (Santa Clara, Calif.). The models resized to 128 × 128 × 32 voxels had 3,995,874 parameters and a mean time of 0.0023 s; when resized to 256 × 256 × 64 voxels, they had 13,471,362 parameters and a mean time of 0.0107 s. The other implantation details are presented in Supplemental File.

For the input of the models, we preprocessed the input DWI images with different normalization and resize schemes. Models A–D were based on NIHSS scores at admission, and Models E–H were based on NIHSS scores on Day 7 of hospitalization (Table 2). For the output of the model, a binary classification for the stage of NIHSS (stage 1 or 2) was conducted. In the test set, we assessed NIHSS scores at admission and on Day 7 of hospitalization and classified them. The same preprocessing methods were adopted as those of the training set. The processed images were imported into the model for classification. The values of AUC, sensitivity, and specificity were evaluated.

### 2.6. Statistical Analysis

*Python* (version 3.8.5; Python Software Foundation, Beaverton, OR, USA.) was used for statistical analysis. Two-sample *t*-tests and chi-square tests were used to compare the clinical information of the training and test sets. The DeLong test was used to compare the performance of the different models. *p*-Values less than 0.05 were considered statistically significant.

## 3. Results

### 3.1. Subjects’ Clinical Information

In total, 711 patients (mean age = 66.02 ± 11.22 years; 538 anterior circulation strokes; 237 women) were enrolled in the training and validation sets. Another 140 patients (mean age = 65.00 ± 10.26 years; 113 anterior circulation strokes; 50 women) were enrolled in the test set. The clinical information of the included patients is presented in Table 3. Age, gender, and lesion location had no significant differences between the training and validation set and the test set. Of the patients of the training and test sets, 393 and 48, respectively, had NIHSS scores less than 5, and the other 318 patients of the training set and 92 patients of the test set had NIHSS scores greater than or equal to 5. The distribution of the classification of NIHSS was significantly different at the time of hospitalization between the training and validation set and the test set (*p* < 0.01). On Day 7 of hospitalization, 445 patients of the training set and 94 patients of the test set had NIHSS scores less than 5, and the other 268 patients of the training set and 46 patients of the test set had NIHSS scores greater than or equal to 5. The distribution of the classification of NIHSS has no significant difference on Day 7 of hospitalization between the training and validation set and the test set (*p* > 0.05).

### 3.2. Classification at Admission

**Figure 4:**
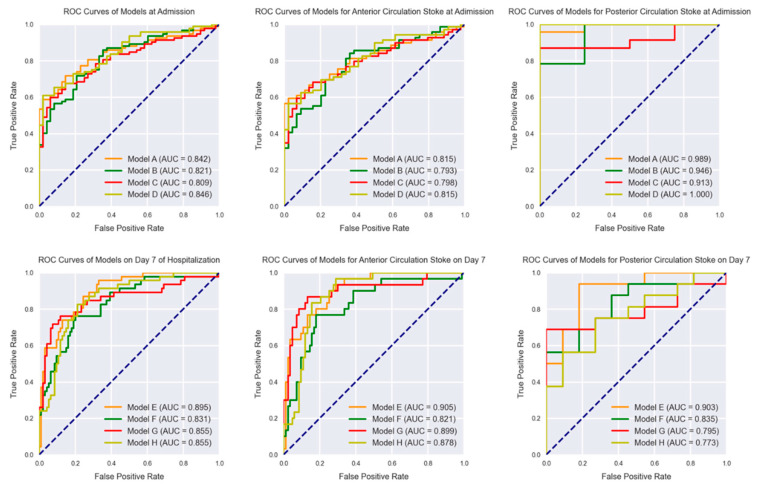
ROC curves of the proposed models in test set for the NIHSS stage predicted at admission, on Day 7 of hospitalization, and at different circulation locations (Day 7 of hospitalization).

With different normalization and resizing schemes, the CNN models based on NIHSS scores at admission had AUC values of 0.809–0.846 (Figure 4 and Table 4). Model D normalized with the z-score and resized to 256 × 256 × 64 voxels the had best AUC of 0.846 (95% CI, 0.776–0.902) with a sensitivity of 60.9% (95% CI, 50.1–70.9%) and a specificity of 97.9% (95% CI, 88.9–99.9%). The DeLong test showed no significant statistical difference between the models based on NIHSS scores at admission (*p* > 0.05).

### 3.3. Classification on Day 7 of Hospitalization

With different normalization and resizing schemes, the CNN models E–G based on NIHSS scores on Day 7 of hospitalization had AUC values of 0.831–0.895 on the test set (Figure 4 and Table 4). Model E normalized by the maximum–minimum and resized to 128 × 128 × 32 voxels achieved the highest AUC value of 0.895 (95% CI, 0.832–0.940), with a sensitivity of 95.7% (95% CI, 88.5–99.9%) and a specificity of 67.0% (95% CI, 56.6–76.4%). Since the AUCs varied among different models, the DeLong test also showed no significant statistical differences between the models based on the NIHSS scores on Day 7 of hospitalization (*p* > 0.05).

### 3.4. Classification of IS Based on Different Circulations

We further analyzed the performance of our predictive models in anterior and posterior circulation stroke. For predicting the NIHSS stage at admission, models A–D achieved AUC values from 0.793 to 0.815 (Table 5). Model A had the best performance and had an AUC value of 0.815 (95% CI, 0.731–0.881), with a sensitivity of 59.4% (95% CI, 46.9–71.1%) and a specificity of 97.7% (95% CI, 88.0–99.9%) (Table 5). When ischemic stroke occurred in the posterior circulation, the models achieved AUC values from 0.946 to 1.000. Model D predicted all of the cases of posterior circulation stroke correctly (Table 6). The AUCs were higher in predicting the NIHSS stage in posterior circulation stroke.

For predicting the NIHSS stage on Day 7 of hospitalization, models E–H had AUC values from 0.821 to 0.905 in predicting the severity of anterior circulation stroke, and Model E achieved the best AUC value of 0.905 (95% CI, 0.836–0.952), with a sensitivity of 90.0% (95% CI, 73.5–97.9%) and a specificity of 74.7% (95% CI, 64.0–83.6%) (Table 5). In the posterior circulation stroke group, the models had AUC values from 0.773 to 0.903 (Table 6), and Model E performed the best with an AUC value of 0.903 (95% CI, 0.727–0.983), a sensitivity of 93.7% (95% CI, 69.8–99.8%), and a specificity of 81.8% (95% CI, 48.2–97.7%). The AUCs were relatively higher in predicting the NIHSS stage on Day 7 of hospitalization in anterior circulation stroke and lower in posterior circulation stroke. The DeLong test also showed no significant statistical differences between the models based on the NIHSS scores on Day 7 of hospitalization (*p* > 0.05).

## 4. Discussion

In this study, we presented a CNN-based classification framework that can preliminarily evaluate the severity of neurological impairment caused by IS, and compared the performance of different time points for NIHSS evaluation, as well as different image preprocessing procedures. Our proposed model achieved better performance in predicting the NIHSS score on Day 7 of hospitalization than that at admission (best AUC 0.895 vs. 0.846). The model also obtained promising performance in subgroup analysis for predicting the NIHSS score on Day 7 of hospitalization, i.e., anterior and posterior circulation stroke, with the best AUCs of 0.905 and 0.903, respectively.

At present, the modified Rankin scale (mRS) and Barthel Index (BI) are mostly used in clinical practice to evaluate the outcome of ischemic patients, and these scales mainly focus on the disability of ischemic patients [24] and self-care ability. In contrast, the NIHSS score is more comprehensive for the neural severity assessment of ischemic patients. Meanwhile, the long-term outcome is not only related to the neural severity but also influenced by other clinical characteristics [25]. The NIHSS has high reliability and validity for the evaluation of neurological function in patients with stroke, and this scale is effective for judging the changes in patients’ condition [4,26]. The validity of the NIHSS varies across time points and increases with the progression of the disease [4,27]. Cai [27] found that mRS, Glasgow Outcome Scale (GOS), BI, and Stroke-Specific Quality of Life (SS-QOL) scores had higher Pearson correlation coefficients with NIHSS scores measured on admission (0.475–0.572) than with NIHSS scores measured on Day 7 of hospitalization (0.592–0.678), showing that the predictive validity of the NIHSS on Day 7 of hospitalization was higher at admission. Similarly, our study showed that the CNN models performed better on Day 7 data than on admission data; in particular, the sensitivity was higher on Day 7. The neurological impairment in acute ischemic stroke is mainly caused by the ischemic and ischemic penumbra. Study [28] showed that 52.4% of patients have DWI/NIHSS mismatch (DNE) because of the existence of ischemic penumbra, and patients benefit from intravenous thrombolysis within six hours of stroke onset [29]. A study on nonhuman primates showed that DNE can last up to 48 h after stroke onset [30]. This may explain the results in terms of the models obtaining better performance in predicting the NIHSS stage on Day 7 of hospitalization than that at admission. At present, DNE is determined mainly by whether the NIHSS score matches the ASPECTS score. In the current study, we developed a 3D-CNN model based on NIHSS to predict the neurological impairment of ischemic patients and classified the severity of neurological impairment by DWI images. The 3D-CNN model can determine whether there is a mismatch between the ischemic core and neurological impairment in patients with acute stroke and can help clinicians to decide on whether vascular recanalization treatment is required in ischemic stroke.

A 3D-CNN can statistically analyze and classify images at the voxel level. This type of model outperforms traditional image analysis methods and has been widely used in medical image analysis in recent years. However, the diversity of MRI scanners and scanning parameters leads to uneven imaging quality and uneven signal intensity, and image standardization is difficult. To identify the best model, we used different preprocessing methods. Two current mainstream image standardization methods, namely, z-score and minimum–maximum normalization [31], were tested for use with this model, and the images were reconstructed with various numbers of voxels. We found that the model performed best when the images were normalized by z-scores and resized to 128 × 128 × 32 voxels. DWI has a lower spatial resolution than other MRI sequences. Our model performed best at a relatively low voxel count; in contrast, other CNN studies have selected higher spatial resolution levels. Although several models performed better, the DeLong test obtained no statistical differences, indicating that a future study is needed to verify the circumstances.

NIHSS scores are more sensitive in anterior than posterior circulation stroke because some signs of acute posterior circulation infarction, such as somatic dyskinesia, nystagmus, and Horner syndrome, are not included in the NIHSS [32]. We further classified patients’ Day 7 NIHSS scores according to the location of the stroke and found that the model was more sensitive in anterior circulation stroke than in posterior circulation stroke, which is consistent with reports in the literature. At the same time, our model also had good performance in posterior circulation stroke; however, it warrants further study with larger sample size.

In patients undergoing acute imaging to evaluate the severity of acute ischemic stroke using DWI–ASPECTS values, MRI is mainly used to evaluate whether there is severe vascular occlusion, where an ASPECTS score of 7 or more represents a high risk of vascular occlusion [33]; meanwhile, the infarct volume can affect prognosis. Jiang’s study showed a strong correlation between the two aspects [34]. At present, there are few studies on the automatic classification of ischemic stroke based on medical images. Ding’s study found that 3D-CNN models can successfully predict the functional outcomes in patients with brainstem infarction with a high AUC of 0.975 [20]. However, in general, there is a lack of effective evaluation methods for ischemic stroke severity based on imaging examinations [35]. In the present study, DWI scans of ischemic patients were used to develop a preliminary 3D-CNN model, and this model successfully classified patients according to their NIHSS scores, illustrating that it is feasible to grade the severity of acute ischemic stroke based on DWI data using a CNN. Wong’s study [13] developed a sematic segmentation model based on U-Net and grouped convolutions and achieved a Dice score of 0.85 in testing. Furthermore, the AUC outcome prediction using stroke volume in 30 refined brain regions based on relevant mRS areas adjusted for clinical variables was 0.80 with an accuracy of 0.75. Another study [36] combined radiological and clinical baseline data for outcome prediction, finding that there was no significant improvement by combining image data with clinical data for mRS prediction, with an AUC of 0.81 vs. 0.80 and above using clinical data only. The NIHSS score was used as a classification of neurological impairment, avoiding interference of other factors affecting the outcome. The infarcted core showed a high signal in the DWI images, which was directly related to the severity of neurological impairment. We used DWI images to train the model, which performed better than that reported in the current literature, and proved that a 3D-CNN can effectively classify the neurological impairment of patients with ischemic stroke by DWI images, affording it broad prospects in clinical application.

The current study has several limitations. First, we did not investigate the performance of deep learning in predicting the functional outcomes of these patients. It is also crucial in clinical practice, and we consider it a future direction. Second, the image parameters were not uniform. This may have compromised the generalizability and stability of our model. However, we adopted preprocessing techniques to maximize the reduction in variability. Finally, the single-center collected sample size was still too small and uneven for deep learning methods. Larger sample size from multi-center institutions is needed to further confirm the efficiency of our model.

## 5. Conclusions

Our proposed 3D-CNN model can effectively predict the NIHSS score of IS using only DWI images and performs better for predicting the NIHSS scores on Day 7 of hospitalization. The model also obtained promising performance in strokes of different circulations. The proposed model can be applied to efficiently evaluate the severity of IS and potentially guide clinical decision making.

## Figures and Tables

**Table 1:** The architecture of the proposed 3D-CNN model.

Model	Type	Filter Size	Number of Filters	Stride
Layer 1	Conv1 + Maximum Pooling	3 × 3 × 3	16	(1, 1, 1)
Layer 2	Conv2 + Maximum Pooling	3 × 3 × 3	32	(2, 2, 2)
Layer 3	Conv3	3 × 3 × 3	64	(1, 1, 1)
Layer 4	Conv4 + Maximum Pooling	3 × 3 × 3	64	(2, 2, 2)
Layer 5	Conv6	3 × 3 × 3	96	(1, 1, 1)
Layer 6	Conv6 + Maximum Pooling	3 × 3 × 3	96	(2, 2, 2)
Layer 7	Conv7	3 × 3 × 3	128	(1, 1, 1)
Layer 8	Conv8 + Maximum Pooling	3 × 3 × 3	128	(2, 2, 2)
Layer 9	FC1	-	-	-
Layer 10	FC2	-	-	-
Layer 11	FC3 (SoftMax)	-	-	-

Conv—convolutional layer; FC—fully connected layer.

**Table 2:** The detailed information for different proposed models with different preprocessing strategies and predicted NIHSS stages.

	Predicted NIHSS Stage	Normalization	Voxels
Model A	Admission	Maximum–minimum	128 × 128 × 32
Model B	Admission	Maximum–minimum	256 × 256 × 64
Model C	Admission	Z-score	128 × 128 × 32
Model D	Admission	Z-score	256 × 256 × 64
Model E	Hospital Day 7	Maximum–minimum	128 × 128 × 32
Model F	Hospital Day 7	Maximum–minimum	256 × 256 × 64
Model G	Hospital Day 7	Z-score	128 × 128 × 32
Model H	Hospital Day 7	Z-score	256 × 256 × 64

**Table 3:** Demographics, location, and class distribution of the included patients.

Characteristics	Training and Validation Sets	Test Set	*p*-Value
Sample capacity	711	140	
Age (years) ^a^	66.02 ± 11.22	65.00 ± 10.26	0.31
Women (%) ^b^	33.1 (237)	35.7 (50)	0.65
Anterior circulation (%)	80.9 (538)	82.9 (113)	0.08
Posterior circulation (%)	25.2 (173)	25.0 (27)	0.83
NIHSS (0 days) <5 (%)	55.3 (393)	34.2 (48)	<0.01
NIHSS (0 days) ≥5 (%)	44.7 (318)	65.7 (92)	<0.01
NIHSS (7 days) <5 (%)	62.7 (445)	67.1 (94)	0.35
NIHSS (7 days) ≥5 (%)	37.3 (268)	32.9 (46)	0.32

^a^ Shown as the mean ± SD. ^b^ Shown as the percentage (number of cases).

**Table 4:** The performance of the models in the test set.

Model	AUC	Sensitivity	Specificity
Model A	0.842 (0.771–0.898)	71.7% (64.1–80.6%)	77.1% (62.7–88.0%)
Model B	0.821 (0.747–0.881)	71.7% (61.4–80.6%)	79.2% (65.0–98.5%)
Model C	0.809 (0.734–0.871)	59.8% (49.0–64.8%)	93.7% (82.8–98.7%)
Model D	0.846 (0.776–0.902)	60.9% (50.1–70.9%)	97.9% (88.9–99.9%)
Model E	0.895 (0.832–0.940)	95.7% (88.5–99.9%)	67.0% (56.6–76.4%)
Model F	0.831 (0.759–0.889)	76.1% (61.2–87.4%)	79.8% (70.2–87.4%)
Model G	0.855 (0.785–0.908)	76.1% (61.2–87.4%)	88.3% (80.0–94.0%)
Model H	0.855 (0.758–0.909)	82.6% (68.6–92.2%)	78.7% (69.1–86.5%)

Models A–D—predicting the NIHSS stage at admission; models E–H—predicting the NIHSS stage on Day 7 of hospitalization. The 95% confidence intervals are presented in brackets.

**Table 5:** The performance of the models in test set in anterior circulation stroke.

Model	AUC	Sensitivity	Specificity
Model A	0.815 (0.731–0.881)	59.4% (46.9–71.1%)	97.7% (88.0–99.9%)
Model B	0.793 (0.707–0.886)	84.1% (77.3–91.8%)	63.6% (47.8–77.6%)
Model C	0.798 (0.712–0.867)	59.4% (46.9–71.1%)	93.2% (81.3–98.6%)
Model D	0.815 (0.731–0.881)	56.5% (44.0–68.4%)	97.7% (88.0–99.9%)
Model E	0.905 (0.836–0.952)	90.0% (73.5–97.9%)	74.7% (64.0–83.6%)
Model F	0.821 (0.738–0.887)	76.7% (57.7–90.1%)	81.9% (72.0–89.5%)
Model G	0.899 (0.828–0.948)	86.7% (69.3–96.2%)	86.7% (77.5–93.2%)
Model H	0.878 (0.803–0.932)	96.7% (82.8–99.9%)	72.7% (39.0–94.0%)

Models A–D—predicting the NIHSS stage at admission; models E–H—predicting the NIHSS stage on Day 7 of hospitalization. The 95% confidence intervals are presented in brackets.

**Table 6:** The performance of the models in the test set in posterior circulation stroke.

Model	AUC	Sensitivity	Specificity
Model A	0.989 (0.853–1.000)	78.3% (56.3–92.5%)	100.0% (39.8–100.0%)
Model B	0.989 (0.853–1.000)	95.6% (77.8–99.9%)	100.0% (39.8–100.0%)
Model C	0.946 (0.785–0.996)	78.3% (56.3–92.5%)	100.0% (39.8–100.0%)
Model D	1.000 (0.872–1.000)	100.0% (85.2–100.0%)	100.0% (39.8–100.0%)
Model E	0.903 (0.727–0.983)	93.7% (69.8–99.8%)	81.8% (48.2–97.7%)
Model F	0.835 (0.643–0.949)	56.2% (29.2–80.2%)	100.0% (71.5–100.0%)
Model G	0.899 (0.828–0.948)	86.7% (69.3–96.2%)	72.7% (39.0–94.0%)
Model H	0.773 (0.572–0.910)	75.0% (47.6–92.7%)	72.7% (39.0–94.0%)

Models A–D—predicting the NIHSS stage at admission; models E–H—predicting the NIHSS stage on Day 7 of hospitalization. The 95% confidence intervals are presented in brackets.

## Data Availability

The authors confirm that the data supporting the findings of this study are available within the article [and/or] its supplementary materials.

## Supplementary Materials

The following supporting information can be downloaded at: https://www.mdpi.com/article/10.3390/jcm11144008/s1.
