# Supplementary material for: Predicting the Severity of Neurological Impairment Caused by Ischemic Stroke Using Deep Learning Based on Diffusion-Weighted Images
Source: J Clin Med. 2022 Jul 11;11(14):4008. doi: 10.3390/jcm11144008 (PMC9325315; doi:10.3390/jcm11144008)
Supplement: Supplementary file 1 [file jcm-11-04008-s001.zip › jcm-1706941-supplementary.pdf]

1. Training details
   All the models with different preprocessing methods and based on different time point were trained by 3D-CNN, and there was no gradient explosion during the training process. After 100 epochs of training, the loss could be reduced to close to zero, which proved that the models had been fully trained, and the best models with the highest accuracies on validation data were selected. During the training process, Model C achieved a best accuracy of 79.4% in models A~D based on NIHSS scores at admission, Model E and F achieved accuracy of 83.8% in models E~H based on NIHSS scores on day 7 of hospitalization (Figure S1).

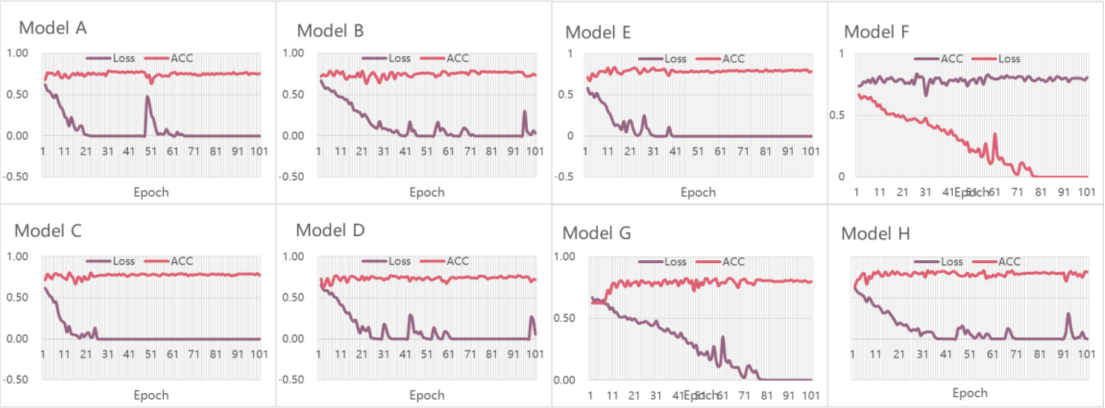

Figure S1. The training information about loss and accuracies of the 8 proposed models.

2. Comparison analysis with other networks
   We added comparison analysis with other networks, like Desnet121, Resnet 18 and Resnext. The results showed that the performance of our proposed models outperformed others (Figures S2~S4, Table S1). The DWI images were resized to 128×128×32 voxels, and normalized with Max-min.

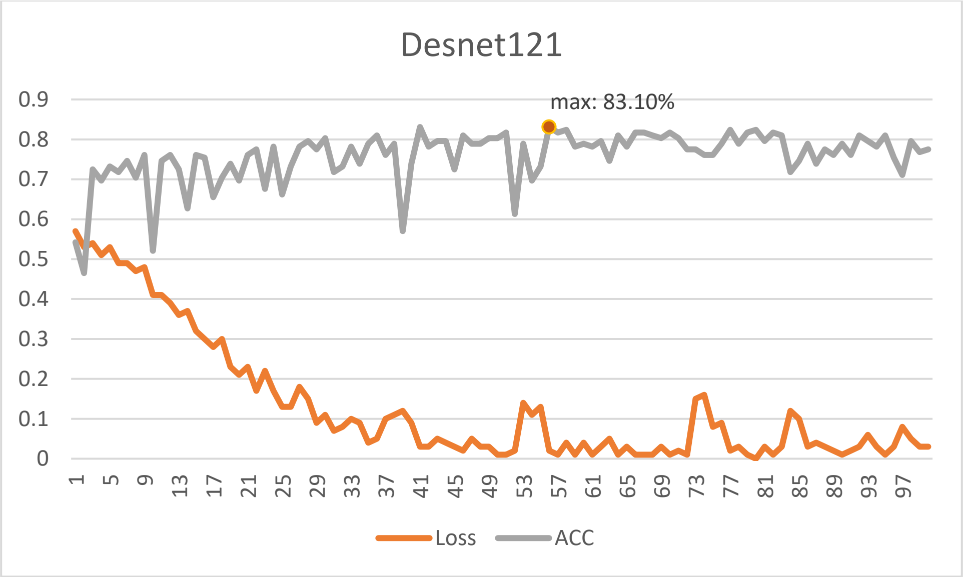

Figure S2. The training information of model based on Desnet121, with an accuracy of 77.8% on test set.

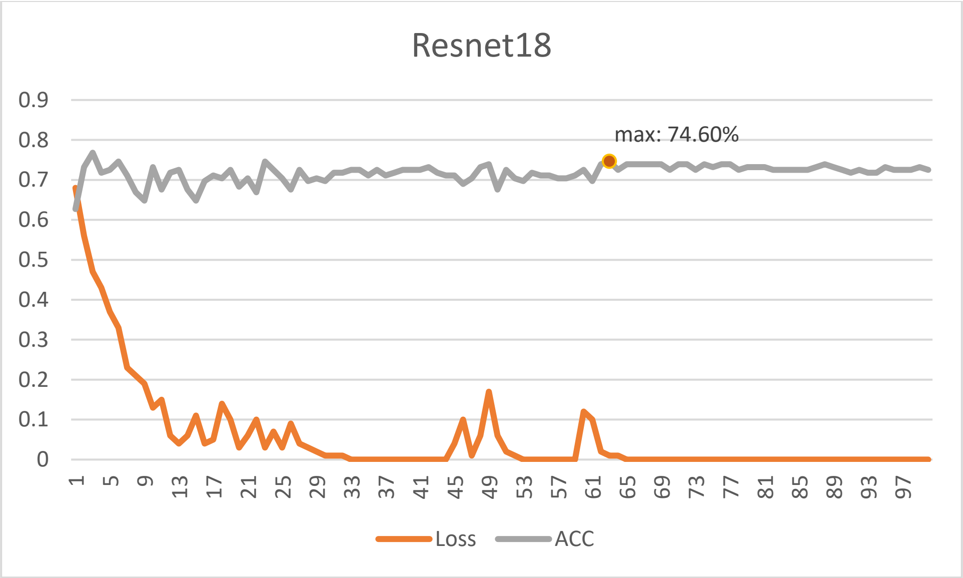

Figure S3. The training information of model based on Resnet18, with an accuracy of 73.6% on test set.

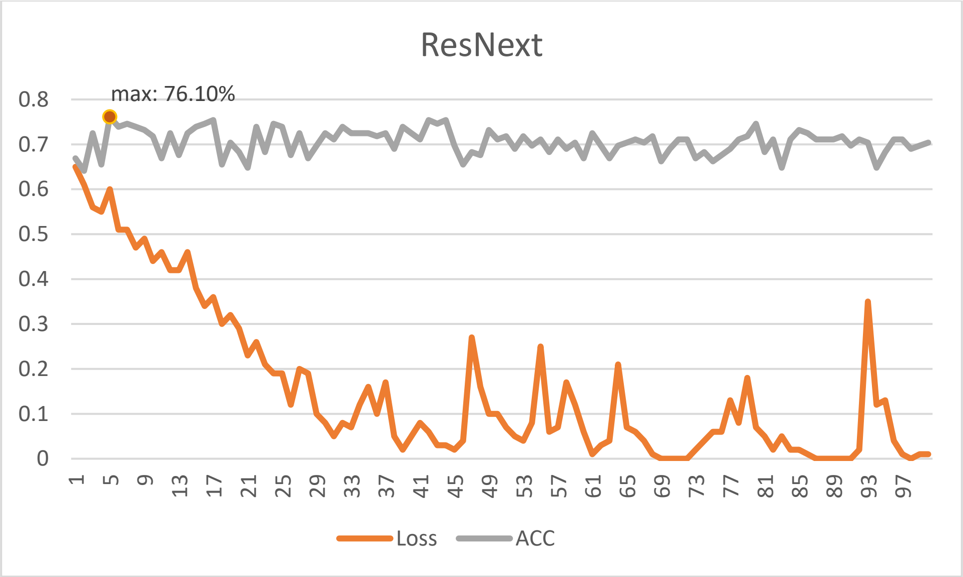

Figure S4. The training information of model based on Resnet18, with an accuracy of 73.6% on test set.

Table S1. ACC values of varies models on validation set and test set

Net	ACC on Validation set	ACC on test set
Desnet121	83.1%	77.9%
Resnet18	74.6%	73.6%
ResNext	76.1%	73.6%
VGG (Model E)	83.8%	81.4%
